# Late Presentation of Unsafe Abortion after 5 Years of Procedure

**DOI:** 10.1155/2014/456017

**Published:** 2014-02-05

**Authors:** Prasanta Kumar Nayak, Subarna Mitra, Alaganandam Padma, Sarita Agrawal

**Affiliations:** ^1^Department of Obstetrics and Gynaecology, All India Institute of Medical Sciences, Tatibandh, Raipur 492099, India; ^2^Department of Obstetrics and Gynaecology, Pondicherry Institute of Medical Sciences, Kalapet, Puducherry 605014, India

## Abstract

A majority of the unsafe abortions are performed by untrained birth attendants or quacks leading to complications in a large proportion of these cases. Complications like bowel injury, bladder injury, uterine perforation, and septic abortion are mostly caused by unskilled hands and are detected immediately or within few days of the procedure, owing to the need for tertiary level care. Here we present a very interesting case of unsafe abortion induced by a Ryle's tube in a 32-year-old lady, which was diagnosed five years after the procedure. Considering its atypical presentation, it is the first case of its kind in the literature. The details of the case and its management are described along with appropriate pictures.

## 1. Introduction

World Health Organization (WHO) defines unsafe abortion as a procedure for terminating an unintended pregnancy either by individuals without the necessary skills or in an environment that does not conform to minimum medical standards or both [1]. In India, unsafe abortion is mostly carried out by untrained birth attendants or quacks. Lack of knowledge and requisite skill as well as disregard about asepsis results in various complications like sepsis, uterine perforation, bowel and bladder injury, and vesicovaginal fistula. These occur primarily due to usage of sharp instruments. Rarely, vesicovaginal fistula can also occur because of the use of some chemical substances for criminal abortion [2].

Rubber catheter has been widely used in India as an abortifacient, as mentioned in a case series of 200 midtrimester medical termination of pregnancies (MTPs) in the early eighties [3]. We report an interesting case where a 70 cm long Ryle's tube was used for illegal abortion causing uterine perforation and partial extrusion of the tube into the peritoneal cavity. Surprisingly, there were no complaints from the patient until 5 years had passed.

## 2. Case Report

A 32-year-old lady, P2L2A1, presented to our hospital with complaints of pain abdomen for the last two months and a foreign body protruding from the introitus for the last 15 days. Patient had earlier gone to a local hospital with complaints of continuous dull aching pain in the lower abdomen, wherein ultrasonography (USG) of abdomen and pelvis revealed a tubular and slender foreign body coiled up in the pelvis and probably in the uterine cavity.

Attempts to remove the foreign body vaginally had failed there; hence, the patient was referred to our institute with the partially extruded foreign body hanging from the introitus. A detailed history was taken in our hospital. Her menstrual cycles were regular and normal. She was married for 10 years and had two full term vaginal deliveries, 8 and 6 years back, respectively. Five years back, she had undergone an abortion at around four months of gestation by a quack.

Though the patient was aware that a foreign body had been pushed into her uterus, she did not know the exact nature of the foreign body. After one day of the procedure, the patient had expelled a fleshy mass with usual amount of bleeding and had been reassured by the quack that her pregnancy had been terminated successfully. She resumed her menses after 1 month of the procedure. Surprisingly, there had been no offensive vaginal discharge, dyspareunia, dysmenorrhoea, fever, or any gastrointestinal symptoms for the following 5 years. The fact that she was not conceiving with unprotected intercourse was not worrying the couple since they never wanted a third child. She was not suffering from any medical disorders.

**Figure 1:**
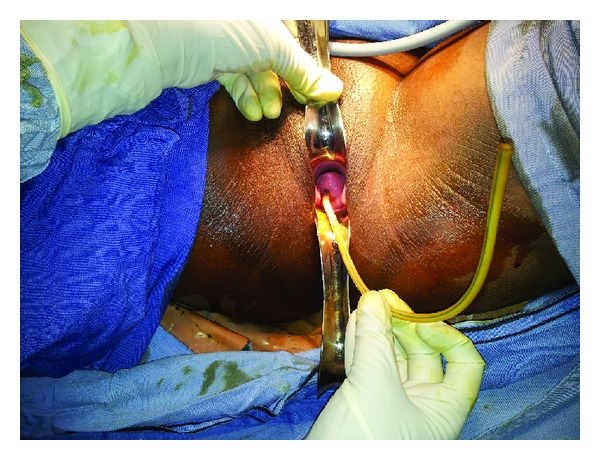
Per speculum examination showing the foreign body protruding out of the cervix.

**Figure 2:**
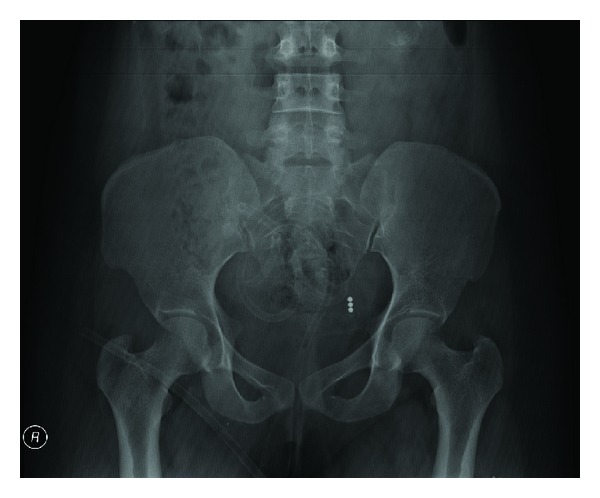
Straight X-ray PA view of the abdomen and pelvis showing the coiled foreign body occupying the pelvis and lower abdomen with three radiolucent beads at one end.

Her general and systemic examination did not reveal any abnormality. There was no abdominal tenderness. A discoloured tubular structure made up of silastic material was seen popping out of the cervix and vagina, which was stuck to the endometrial cavity and caused pain when attempt was made to drag it out (Figure 1). Uterine size was normal. Straight X-ray PA view of the abdomen and pelvis revealed a radiolucent tubular structure with three radiopaque beads at the other end which was coiled inside to occupy the pelvis and lower abdomen (Figure 2).

From this, we inferred that a Ryle's tube must have been used for the abortion, which had perforated the uterus and had been left in situ without the knowledge of the patient and without producing any significant complications for long.

Cystoscopy excluded bladder involvement. Hysteroscopy showed that the tube has pierced through the posterior wall of the uterus and there were no intrauterine adhesions.

**Figure 3:**
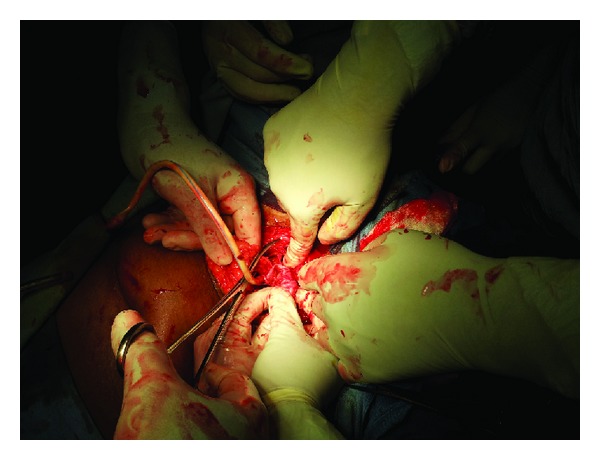
Laparotomy picture showing the dense adhesion of loops of bowel and omentum to the foreign body.

**Figure 4:**
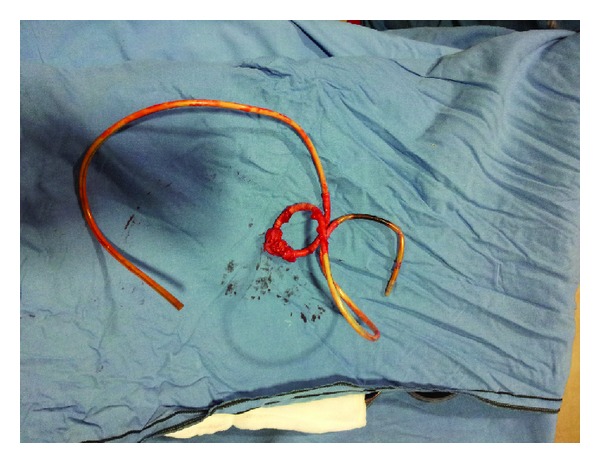
The entire foreign body after its removal.

Laparoscopy revealed that the Ryle's tube had perforated the uterus through the posterior fundal wall and two-thirds of the tube was lying coiled inside the peritoneal cavity. Bowel and omental loops were adherent to the entire length of the intra-abdominal portion of the tube (Figure 3). Laparoscopic adhesiolysis was tried but was difficult because of dense and organized fibrosis. Apprehending bowel injury and excessive bleeding, we decided to switch over to laparotomy. Adhesiolysis was performed carefully and the freed tube was dragged out vaginally (Figure 4). The rent on the uterine fundus was repaired and haemostasis achieved.

Bilateral tubectomy was performed by modified Pomeroy's technique with the prior consent of the patient. Cefotaxime, metronidazole, and gentamicin were administered for 3 days. Postoperative period was uneventful and the patient was discharged on fifth postoperative day.

## 3. Discussion

The literature reveals volumes of research articles, case reports, and letters to the editor on unsafe abortion. All these articles deliver a common message that MTP done by unskilled hands, without taking aseptic measures, leads to severe health consequences ranging from visceral injury, genital fistula, and septicaemia to death of the patient. Annually around 50 million women undergo abortions, out of which about 19 to 20 million of them are unsafe abortions [4]. Approximately, 68,000 women die annually, because of abortion and a high proportion of these deaths are due to illegal and unsafe abortion [5]. According to WHO, every 8 minutes, a woman from a developing nation is dying due to complications arising from unsafe abortion, making it one of the important yet preventable causes of maternal mortality (13%) [6]. Objects used for abortion are knitting needles, sticks, roots of plant, or even a Bougie (large rubber catheter) [5]. These complications manifest immediately after the procedure or within few days [7, 8]. But in this case, the fact that a 70 cm long Ryle's tube continued to adapt silently inside the human body for five long years without the knowledge of the patient is an unusual occurrence. Ryle's tube, which is also known as nasogastric tube, is made up of polyurethane and is used for feeding and administering drugs.

Remarkably and incredibly, typical features of an intrauterine and intra-abdominal foreign body, like menstrual irregularity, discharge per vaginum, chronic pelvic pain, fever, vomiting, subacute intestinal obstruction, and so forth, were all absent. Possible explanation for this can be the fact that Ryle's tube is made up of inert material and the quack may have used some antibiotics to avoid infection. It is mentioned in the literature that feeding tube can dislodge accidentally from the gastrointestinal tract and it may either remain asymptomatic or result in small bowel obstruction [9]. It is possible that the initial perforation was partial through a relatively less vascular part of the uterine wall, which would have caused abdominal pain and bleeding per vaginum but was assumed to be due to abortion. Subsequent uterine involution and myometrial contractions would have made the perforation complete. Slow entry of this inert tube probably did not produce any injury to bowel or omentum. The intra-abdominal portion of the tube produced organised fibrosis. Low educational status and no desire for further issue never made the couple worry about secondary infertility. This infertility could be due to an intrauterine device (IUD) like effect of the foreign body or due to tubal block resulting from peritubal adhesions.

There are several learning points from this case. Unsafe abortions should be discouraged. The risk of complications and death from unsafe abortion is inversely related to the provider's skill, conditions for performing the procedure, and availability of appropriate equipment. Any uterine instrumentation should always be carried out in the hospital by a qualified person and the patient should be informed about the details of the procedure. Complete expulsion of the products of conception should be ensured, preferably by USG.

Criminal abortions are rampant in developing countries but get highlighted only if unsuccessful or complicated [10]. Government should take active interest in promoting social awareness on the dangers of unsafe abortion and organize effective campaigns and programmes in every nook and corner of the country.

## 4. Conclusion

Many unsafe abortions are not reported because of presumed successful termination, only to be detected later with some complications. This makes it a major factor in maternal morbidity and mortality. Hence, it is the combined responsibility of the government and health care providers to provide easy access to safe abortion services as well as to create social awareness regarding safe and legal abortion methods.
